# Expanding the phenotype of Wolfram syndrome: adult presentation with a novel *WFS1* variant

**DOI:** 10.1210/jcemcr/luag066

**Published:** 2026-05-05

**Authors:** Pulkit Mehrotra, Vengadakrishnan, Nisha Dubey

**Affiliations:** Department of General Medicine, Sri Ramachandra Medical College, Porur 600116, India; Department of General Medicine, Sri Ramachandra Medical College, Porur 600116, India; Department of General Medicine, Sri Ramachandra Medical College, Porur 600116, India

**Keywords:** Wolfram syndrome type 1 (WS1), DIDMOAD spectrum, optical coherence tomography (OCT), magnetic resonance imaging (MRI), genetic confirmation, sodium valproate

## Abstract

Wolfram syndrome type 1 (WS1) is a rare autosomal recessive disorder involving diabetes mellitus, optic atrophy, and neurodegeneration, caused by biallelic *WFS1* mutations. Though typically diagnosed in childhood, adult-onset cases may be missed due to variable symptom onset. We describe a 38-year-old woman with early-onset insulin-requiring, autoantibody-negative diabetes, progressive visual loss due to optic atrophy, bilateral sensorineural hearing loss, secondary amenorrhea with hyperprolactinemia, and arginine-vasopressin (AVP) deficiency. Magnetic resonance imaging (MRI) demonstrated atrophy of the optic nerves/chiasm and cerebellum. Genetic testing revealed a novel homozygous exon 8 *WFS1* loss-of-function variant, which was classified as pathogenic. This case underscores the need for heightened clinical suspicion, imaging-based markers, and timely genetic testing to diagnose Wolfram syndrome in adult patients and to guide their management and eligibility for emerging trials.

## Introduction

Wolfram syndrome type 1 (WS1) arises from biallelic variants in the *WFS1* gene encoding wolframin, an endoplasmic reticulum (ER) transmembrane protein critical for calcium homeostasis and the unfolded protein response. Beta-cell and neuronal vulnerability due to *WFS1* dysfunction underlies the DIDMOAD spectrum [Diabetes Insipidus; Diabetes Mellitus (Juvenile-onset); Optic Atrophy and Deafness (Sensorineural)] of arginine-vasopressin (AVP) deficiency, diabetes mellitus, optic atrophy, and deafness. Most patients develop diabetes in childhood and optic atrophy in adolescence, but adult-onset or adult-diagnosed cases are increasingly recognized due to staggered symptom manifestations and limited awareness in adult clinical settings [1-4]. WS1 is extremely rare, with an estimated prevalence of approximately 1 in 500 000 people worldwide [1, 4]. Here, we report an adult patient with WS1 to expand the phenotypic spectrum and highlight the importance of early recognition and genetic confirmation.

## Case presentation

A 38-year-old woman presented with progressive blurring of vision, gait imbalance, polyuria, polydipsia, secondary amenorrhea, and difficulty hearing. She had been diagnosed with insulin-requiring diabetes mellitus at age 6, with negative pancreatic autoantibodies. Family history was noncontributory, and there was no known consanguinity.

## Diagnostic assessment

**Figure 1:**
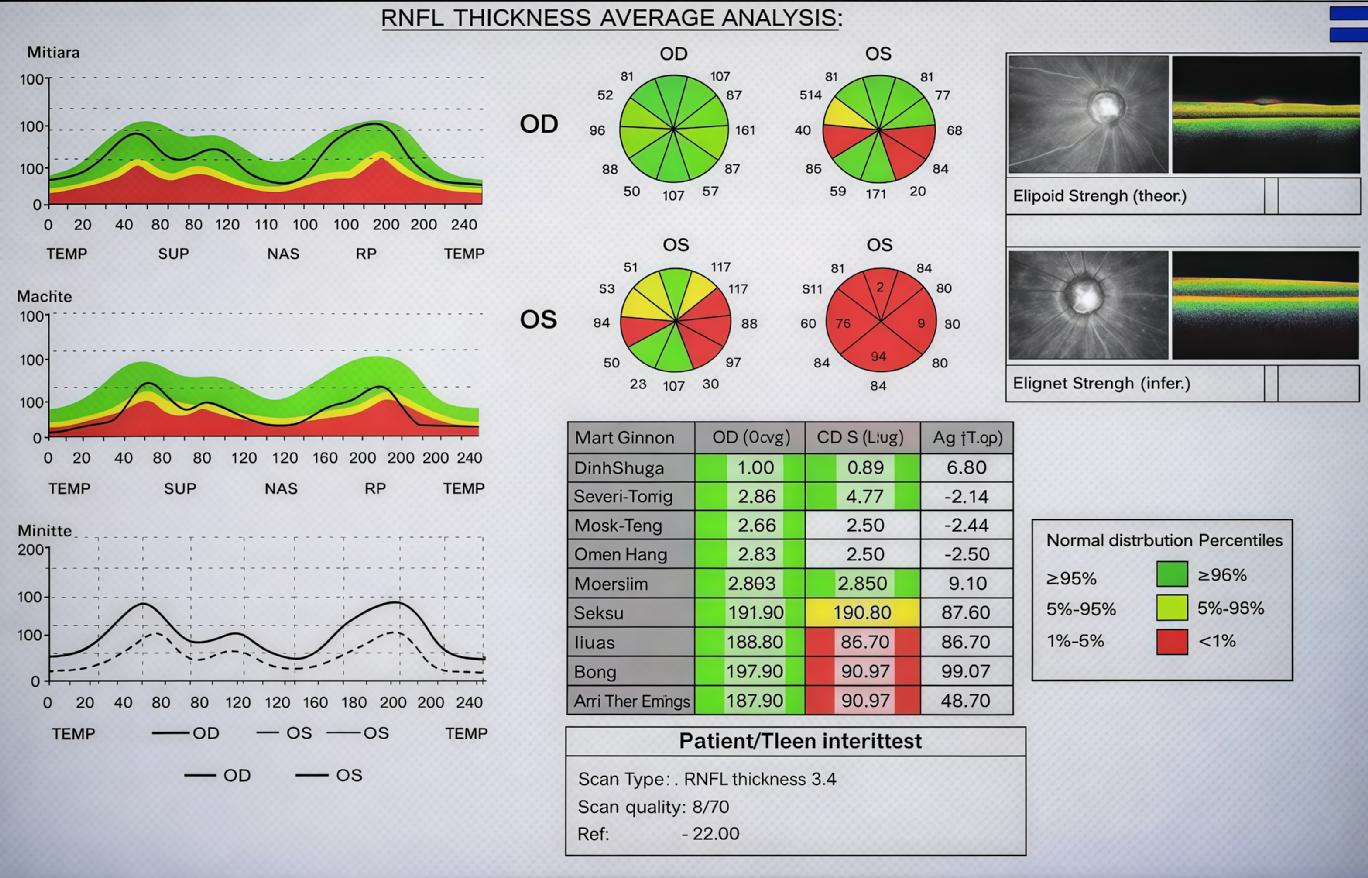
Optical coherence tomography (OCT) report showing retinal nerve fiber layer thinning.

**Figure 2:**
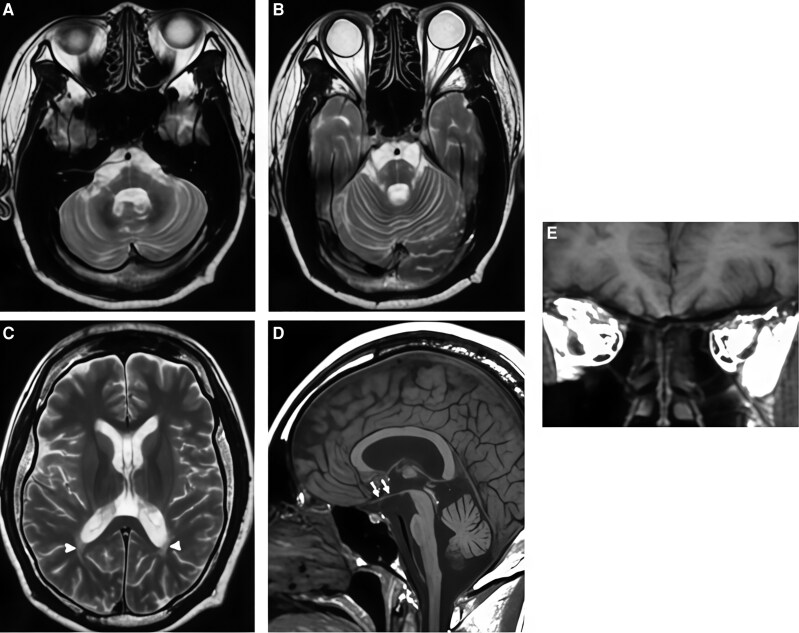
MRI brain showing optic nerve and cerebellar/brainstem atrophy in Wolfram syndrome; (A, B) Axial T2-weighted images at the level of the posterior fossa showing prominent cerebellar atrophy, with thinning of the cerebellar hemispheres and vermis (white arrows). (C) Axial T2-weighted image at the supratentorial level demonstrating mild ventricular prominence with diffuse cerebral volume loss, consistent with generalized cerebral atrophy (white arrowheads). (D) Mid-sagittal T1-weighted image showing marked cerebellar vermian and brainstem (pons and midbrain) atrophy, with thinning of the brainstem contour (white arrows). (E) Coronal image through the orbit demonstrating bilateral optic nerve atrophy, consistent with optic neuropathy (white arrows).

At presentation, glycated hemoglobin (HbA1c) was 8.5% (69 mmol/mol) (reference range, ≤5.6% [≤38 mmol/mol]). Fasting plasma glucose was 106 mg/dL (SI: 5.9 mmol/L) (reference range, 70-99 mg/dL [SI: 3.9-5.5 mmol/L]), and 2-hour postprandial plasma glucose was 214 mg/dL (SI: 11.9 mmol/L) (reference range, <140 mg/dL [SI: <7.8 mmol/L]). Serum sodium was 139 mmol/L (reference range, 135-145 mmol/L), and serum potassium was 3.5 mmol/L (reference range, 3.5-5.0 mmol/L). Serum creatinine was 0.33 mg/dL (SI: 29 µmol/L) (reference range, 0.5-1.0 mg/dL [SI: 45-90 µmol/L]). Endocrine evaluation demonstrated marked hyperprolactinemia, with a serum prolactin concentration of 120.7 ng/mL (SI: 120.7 µg/L) (reference range, 5-25 ng/mL [SI: 5-25 µg/L]). Gonadotropin levels were suppressed, with luteinizing hormone (LH) 0.18 IU/L (reference range, 1.9-12.5 IU/L, follicular phase) and follicle-stimulating hormone (FSH) 2.51 mIU/mL (reference range, 2.5-10.2 mIU/mL, follicular phase). Serum estradiol was 77.7 pg/mL (SI: 285 pmol/L) (reference range, 19-140 pg/mL [SI: 70-514 pmol/L]), consistent with hypogonadotropic hypogonadism. Baseline biochemical and hormonal investigations at presentation are summarized in Table 1. Ophthalmologic examination showed bilateral optic disc pallor, and optical coherence tomography demonstrated significant retinal nerve fiber layer thinning consistent with optic neuropathy (Fig. 1). Genetic testing (targeted gene panel sequencing, confirmed by Sanger sequencing) identified a homozygous loss-of-function variant in *WFS1* (exon 8). This variant is absent from population databases and predicted deleterious in silico; it was classified as pathogenic according to ACMG/AMP (American College of Genetics and Genomics/Association for Molecular Pathology) criteria (fulfilling PVS1, PM2, PP3). Collectively, these findings established the diagnosis of WS1 in the patient. The detailed ACMG/AMP criteria supporting pathogenic classification of the *WFS1* variant are outlined in Table 2. Magnetic resonance imaging (MRI) of the brain revealed marked atrophy of the optic nerves and chiasm, along with significant atrophy of the brainstem and cerebellum (Fig. 2).

**Table 1:** Biochemical and hormonal test results on presentation

Parameter	Result	Reference range
HbA1c	8.5%	≤ 5.6%
Fasting plasma glucose	5.9 mmol/L (106 mg/dL)	3.9-5.5 mmol/L (70-99 mg/dL)
2-hour post-prandial glucose	11.9 mmol/L (214 mg/dL)	< 7.8 mmol/L (< 140 mg/dL)
Serum sodium	139 mmol/L	135-145 mmol/L
Serum potassium	3.5 mmol/L	3.5-5.0 mmol/L
Serum creatinine	29 μmol/L (0.33 mg/dL)	45-90 μmol/L (0.5-1.0 mg/dL)
Serum prolactin	120.7 ng/mL	5-25 ng/mL
Luteinizing hormone (LH)	0.18 IU/L	1.9-12.5 IU/L (follicular)
Follicle-stimulating hormone (FSH)	2.51 mIU/mL	2.5-10.2 mIU/mL (follicular)
Estradiol	77.7 pg/mL	19-140 pg/mL (follicular)

**Table 2:** ACMG/AMP classification of the patient's WFS1 variant

Criterion	Evidence supporting pathogenicity	Interpretation
PVS1 (very strong)	The patient harbours a homozygous exon 8 loss-of-function variant in *WFS1*. Loss-of-function variants in *WFS1* are a well-established mechanism for Wolfram syndrome	Null variants in a gene where loss of function is a known mechanism support pathogenicity
PM2 (moderate)	The variant is absent or extremely rare in population databases such as gnomAD	Absence from controls provides moderate evidence for a deleterious effect
PP3 (supporting)	Multiple in-silico prediction tools suggest a deleterious impact on protein function	Concordant computational evidence lends supporting weight

Overall, the combination of a very strong criterion (PVS1), a moderate criterion (PM2), and supporting evidence (PP3) meets the ACMG/AMP threshold for a pathogenic classification.

## Treatment

The patient was managed with a basal–bolus insulin regimen for glycemic control and intranasal desmopressin for central AVP deficiency. She was also started on combined estrogen–progestin hormone replacement therapy to address hypogonadotropic hypogonadism (secondary amenorrhea). Supportive care measures included bilateral hearing aids, low-vision assistive devices, patient counseling, and coordinated multidisciplinary follow-up with endocrinology, ophthalmology, audiology, and neurology services.

## Outcome and follow-up

After initiating the above treatments, her polyuria and polydipsia improved significantly with desmopressin. She continues to undergo low-vision rehabilitation and regular audiologic evaluations. Ongoing endocrine follow-up has been arranged to monitor her prolactin levels and the potential resumption of menses.

## Discussion

Wolfram syndrome is a rare cause of young-onset diabetes accompanied by progressive neurodegeneration. In an adult patient with insulin-dependent, nonautoimmune diabetes and neurological features, the differential diagnosis includes other syndromic causes of diabetes and deafness. These include maternally inherited diabetes and deafness (MIDD, due to mitochondrial DNA mutations) [1, 5], autosomal dominant optic atrophy with diabetes (for example, caused by *OPA1* variants), and Alström syndrome (due to *ALMS1* mutations, typically with additional cardiometabolic features) [1, 5]. In our patient, the combination of long-standing insulin-requiring diabetes, optic atrophy, sensorineural hearing loss, and central AVP deficiency strongly pointed to WS1, which was confirmed by genetic testing.

WS1 results from loss-of-function mutations in *WFS1*, the gene encoding wolframin. Over 200 pathogenic *WFS1* variants have been described across the gene [5], with exon 8 being a frequent.

hotspot for disease-causing changes. Our patient's homozygous exon 8 variant appears to be novel; we classified it as pathogenic based on ACMG/AMP criteria, and we will submit this variant to a public database to facilitate future reference. Notably, an adult-onset WS1 case with a different *WFS1* mutation (a homozygous in-frame deletion, p.Lys193del) has been reported in the literature [6]. That patient, who presented at age 38 years with similar features, underscores that certain *WFS1* mutations can manifest with later-onset diabetes and milder initial symptoms [6].

Objective clinical testing can aid in recognizing and monitoring WS1. Optical coherence tomography (OCT) provides quantitative evidence of optic neuropathy, typically revealing retinal nerve fiber layer thinning in WS1 patients. Likewise, MRI often demonstrates brainstem and cerebellar atrophy in wolfram syndrome [4], as was observed in our case (Fig. 2, white arrows). Investigational biomarkers such as circulating microRNA profiles have shown correlation with neurological disease severity [7], but these are not yet applicable to routine care.

There is no approved disease-modifying treatment for WS1 at present. Management is supportive and focused on each manifestation (endocrine, ophthalmologic, etc.). Several experimental therapies targeting ER stress and calcium homeostasis are under study. For example, dantrolene sodium, which modulates ER calcium release, demonstrated an acceptable safety profile in a phase Ib/IIa trial in WS1 patients [8] (ClinicalTrials.gov NCT02829268) [9]. Additionally, sodium valproate is being evaluated as a repurposed therapy in an ongoing randomized trial (TREATWOLFRAM) [10]. Early genetic confirmation in suspected WS1 is critical, as it enables referral of patients to such clinical trials, as well as appropriate family counseling, reproductive planning, and specialized multidisciplinary care.

Importantly, this case illustrates the consequences of a delayed diagnosis. When WS1 is only recognized in adulthood, the patient may have already suffered irreversible vision loss, hearing impairment, and other neurological damage by the time of diagnosis. Furthermore, a late diagnosis means missed opportunities for earlier interventions or inclusion in clinical trials at a younger age. Heightening awareness of WS1 among adult endocrinologists and ophthalmologists is therefore essential to facilitate timely diagnosis, which can improve long-term management and outcomes.

## Learning points

- Adult-onset WS1 should be suspected in patients with insulin-dependent, autoantibody-negative diabetes who develop optic atrophy, sensorineural hearing loss, or unexplained arginine-vasopressin (AVP) deficiency.
- Objective tests (OCT, brain MRI) can document retinal nerve fiber layer loss and brainstem–cerebellar atrophy, supporting the diagnosis.
- Early *WFS1* genetic testing is crucial; any identified variant should be assessed using ACMG/AMP criteria and followed by genetic counseling and cascade testing.
- Although no approved disease-modifying therapy exists, eligible patients should be referred to clinical trials of treatments targeting endoplasmic reticulum stress and calcium dysregulation pathways.

## Data Availability

Some or all datasets generated during and/or analyzed during the current study are not publicly available but are available from the corresponding author on reasonable request.
